# Furious snarling: Teeth-exposure and anxiety-related attentional bias towards angry faces

**DOI:** 10.1371/journal.pone.0207695

**Published:** 2018-11-27

**Authors:** Benedikt Emanuel Wirth, Dirk Wentura

**Affiliations:** Department of Psychology, Saarland University, Saarbrücken, Germany; University of the West of Scotland, UNITED KINGDOM

## Abstract

Dot-probe studies consistently show that high trait anxious individuals have an attentional bias towards threatening faces. However, little is known about the influence of perceptual confounds of specific emotional expressions on this effect. Teeth-exposure was recently recognized as an important factor for the occurrence of attentional bias towards angry faces in a closely related paradigm (the face-in-the-crowd paradigm). Therefore, we investigated the effect of exposed teeth on attentional bias towards angry faces in the dot-probe task. Participants (*N* = 74) were asked to classify probe stimuli that were preceded by two simultaneously presented face cues, one angry and the other neutral. Half of the angry faces had exposed teeth, the other half had concealed teeth. Afterwards, participants completed the trait anxiety scale of the STAI. For angry faces with non-exposed teeth, we found the expected positive correlation (*r* = .441) of trait anxiety with the attentional bias score (reaction times for probes replacing the neutral face minus reaction times for probes replacing the angry face). However, we found no influence of trait anxiety on attentional bias towards angry faces with exposed teeth. These results suggest that natural low-level stimulus confounds of emotional faces like exposed teeth can affect the manifestation of anxiety-related attentional biases towards angry faces in the dot-probe task.

## Introduction

Facial expressions are undoubtedly an important social signal in everyday life. In particular, angry faces are considered to be a relevant stimulus in our environment because angry faces convey a signal of imminent threat to the observer that may require immediate action to secure one’s own well-being [1–3]. Therefore, a considerable amount of research has discussed the question of whether angry faces are processed in a prioritized manner. In particular, two paradigms have been applied to investigate this issue. The first paradigm, the face-in-the-crowd paradigm, is a variant of visual search (see [4,5] for descriptions of the basic visual search paradigm). In the face-in-the-crowd paradigm, participants are asked to search for a target face displaying a specific (or merely discrepant) emotion among a set of distractor faces. For example, participants would be presented a crowd of happy faces and would be asked whether there is an angry face (or a non-happy face) among them. In this paradigm, an attentional bias towards threatening stimuli is inferred, given that either two of the following criteria is met: (1) if participants are faster to detect an angry target face among neutral (or positive) distractor faces than to detect a neutral (or positive) target face among angry distractor faces; (2) if increasing the number of distractor faces leads to smaller increases in search times for angry faces among neutral (or positive) distractors than for a neutral (or positive) target among threatening distractors (see [6] for a review article on the paradigm). Numerous studies employing both photographic faces [7–9] and schematic faces [2,10,11] as stimuli have found a search advantage for angry faces compared to faces with different expressions—a finding often referred to as the anger-superiority effect. The occurrence of the anger-superiority effect in face-in-the-crowd studies suggests that humans generally show an attentional bias towards angry faces. One potential explanation for this attentional bias is that during human phylogeny, aggression among conspecifics frequently occurred. Due to this evolutionary pressure, it became necessary to quickly detect and decode social signals of dominance and submission [3].

The second paradigm that has frequently been used to investigate attentional biases towards threatening faces is a variant of spatial cueing (see [12,13] for descriptions of the basic paradigm), namely the dot-probe task. The dot-probe task was originally developed by MacLeod et al. [14] to assess attentional biases towards emotional (especially threatening) words. The dot-probe task has been conducted with a large variety of stimuli, for example emotional words [14,15] and scenes [16–18] or fear-conditioned stimuli [19,20]. Importantly, numerous studies have also investigated attentional bias towards emotional faces (see [21] for an extensive review). In this variant of the paradigm, participants are asked to respond to a probe stimulus that can appear in either of two screen positions (usually left or right off center). The probe is preceded by two face cues that are presented in the potential probe positions, one emotional (usually threatening, e.g., angry) and one neutral. Importantly, the probe position is uncorrelated with the positions of the preceding face cues. An attentional bias towards emotional faces is inferred if participants are faster to respond to probes appearing at the same position as emotional faces than to probes appearing at the same position as neutral faces. The idea here is twofold: First, if emotional faces capture attention, the participant’s attentional focus is already in the right position if the probe appears at the location of the emotional face, which leads to faster reaction times. Second, if attention dwells on an emotional face, problems with disengagement can increase reaction times when the probe appears at the location of the neutral face. Currently, it is still debated whether the dot-probe task measures the former or the latter process—or potentially both processes [22–24].

Meta-analyses have shown that in the dot-probe task, attentional bias towards threatening faces only occurs for anxious participants, but not for non-anxious participants [25,26]. Moreover, a recent qualitative review of 71 dot probe studies [21] shows that the overwhelming majority of dot-probe studies does not find an attentional bias towards threatening faces in non-anxious participants. This finding is consistent with several clinical models of anxiety that claim that attentional bias to threat is a key component of abnormal cognitive processing in anxiety [27–29]. A recent review article argues that the attentional bias might even be causally related to fear and anxiety [30].

Taken together, the face-in-the-crowd paradigm usually finds an attentional bias towards angry faces (as indicated by the anger-superiority effect) in unselected samples (that should be representative of the general population). In contrast, the dot-probe task usually finds an attentional bias towards threatening faces only in anxious participants. Consequently, there is a discrepancy between both paradigms regarding attentional bias towards threatening faces in non-anxious participants. The aim of the present study is to investigate one possible source of this discrepancy: perceptual low-level confounds of faces that can occur naturally in specific emotional expressions.

The issue of perceptual low-level confounds has been discussed extensively in the face-in-the crowd community. It should be noted that even within this community, study results have not always been entirely consistent since a few studies found a search advantage for happy faces instead of an anger-superiority effect [31,32]. A potential explanation for these inconsistent results is that facial expressions often have natural perceptual confounds that are easily detected in a crowd, such as the high luminance of exposed teeth in an angry snarl or in a toothy grin [33], or the high luminance of the exposed sclera in a fearful stare [34]. Consequently, search advantages for emotional expressions might not occur due to the emotional nature of these stimuli, but due to their low-level confounds. Consistent with this idea, Horstmann and Bauland [7] found a search advantage for angry faces and this search advantage also occurred when all facial features except the mouth were removed from the stimuli. Conversely, Calvo and Nummenmaa [35] found a search advantage for happy faces and this search advantage also occurred when only isolated mouths were presented. Since isolated mouths hardly convey any emotional expression, these findings can only be explained by the perceptual properties of the mouth regions of the respective stimuli employed by the two studies. The finding that differences in search efficiency between specific emotional expressions are hardly affected by face inversion also suggests that these differences are caused by isolated facial features and not by a holistic emotional impression conveyed by these faces [36]. Moreover, Savage et al. [37] found a search advantage for angry faces when using a stimulus database with angry faces that are particularly salient relative to other emotional faces contained in the database. In contrast, when they used a stimulus database with relatively salient happy faces, they found a search advantage for happy faces. Additionally, Horstmann et al. [33] showed that search advantages for specific emotions are largely caused by the perceptual saliency of exposed teeth. When happy faces had exposed teeth while angry faces did not, search was more efficient for happy faces. Conversely, when angry faces had exposed teeth while happy faces did not, search was more efficient for angry faces.

These studies show that the face-in-the-crowd paradigm—one of the two paradigms that are mainly used to assess attentional biases towards threatening faces,—is critically affected by perceptual low-level confounds of emotional expressions, such as exposed teeth. To our knowledge, however, no studies have investigated the impact of such confounds on attentional bias in the other paradigm, the dot-probe task. Therefore, the present study aims to investigate the role of exposed teeth in attentional bias towards angry faces in the dot-probe task. It should be noted, however, that there are two critical differences between the face-in-the-crowd paradigm and the dot-probe task. First, while the face stimuli are irrelevant to the participants’ task in the dot-probe paradigm, participants are actively searching for a specific face in the face-in-the-crowd paradigm. Therefore, in the face-in-the-crowd-paradigm, participants can strategically use salient perceptual confounds to facilitate their task and find the target face faster [33]. In contrast, participants have no incentive to strategically attend to salient confounds of the face cues in the dot-probe task. Second, as already mentioned, several reviews and meta-analyses [21,25,26] have shown that the dot-probe task usually finds an attentional bias towards angry faces only in anxious participants (see also [38]).

Nevertheless, perceptual stimulus characteristics like exposed teeth might play a role in anxiety-related attentional bias towards task-irrelevant threatening stimuli in the dot-probe task. According to a review article by Cisler and Koster [39], both bottom-up and top-down processes play a role in anxiety-related attentional biases to threat. Bottom-up processes refer to influences on attention that are caused by the stimulus itself, for example, by its perceptual saliency [40,41]. In contrast, top-down processes refer to influences on attention that are not caused by the stimulus, but by characteristics of the observer, for example his current goals and motivations [42,43]. Cisler and Koster [39] argue that specific stimulus properties can trigger a threat detection mechanism via bottom-up processes. This threat detection mechanism is hyper-sensitive in anxious individuals, which results in facilitated initial allocation of attention to threat (i.e., in an attentional engagement bias). Conversely, deficits in attentional top-down control mediate biases in attentional disengagement. Thus, perceptually salient stimulus properties (like exposed teeth) can potentially affect the manifestation of anxiety-related biases in attentional engagement.

Specifically, there are three potential hypotheses in which way exposed teeth could affect attentional bias towards angry faces in dot-probe studies. First, it is possible that due to their perceptual saliency, exposed teeth capture attention in both anxious and non-anxious participants. For example, numerous basic attention studies employing the additional-singleton paradigm have shown that highly salient stimuli capture visual attention despite being irrelevant to the participants’ task (e.g., [41,44,45]). If exposed teeth are salient enough to directly capture attention (because of their high luminance and contrast), participants should show a general (i.e., anxiety-independent) bias towards angry faces with exposed teeth in the dot-probe task. The occurrence of this general bias would interfere with the detection of the typically found anxiety-related bias. Similar to this rationale, Dodd et al. [46] recently showed that top-down processes can affect the manifestation of anxiety-related bias to threat. Participants performed a face-in-the-crowd task where emotional expressions were either task-relevant or task-irrelevant. When emotional expressions were task-irrelevant, only anxious participants showed a relative bias towards angry compared to happy faces. However, when emotional expressions were task-relevant, both anxious and non-anxious participants showed an attentional bias to emotion and no difference between anxious and non-anxious participants occurred.

Second, it is possible that exposed teeth do not cause a general attentional bias, but that the perceptual heterogeneity between the salient angry face cues with exposed teeth and the less salient neutral face cues with concealed teeth creates noise that reduces the detectability of the anxiety-related attentional bias towards angry faces. Third, it is even possible that exposed teeth cause (or enhance) the typically found correlation between trait anxiety and bias towards threatening faces. Attentional control theory [47–49] claims that anxiety disrupts the balance between stimulus-driven and goal-directed attentional processes to the effect that the influence of bottom-up processes on attention is increased in anxious individuals. Although this bias should be particularly pronounced for threatening stimuli, the theory predicts that any bottom-up influences on attention (e.g., saliency-driven influences) are increased in anxious individuals. For example, Moser et al. [50] showed in a study employing the additional-singleton paradigm that attentional capture by highly salient but task-irrelevant color singletons was larger for anxious than for non-anxious participants (although the color singletons were not threatening or in any way emotional). Thus, perceptually salient components of the face cues in the dot-probe task might affect attention to a larger degree in anxious individuals than in non-anxious individuals.

In order to test these three competing hypotheses, the present study investigated the effect of teeth-exposure on the measurement of attentional bias towards angry faces in the dot-probe task. To this end, we conducted a dot-probe study where teeth-exposure of the angry face cues was experimentally varied.

It should be noted, however, that teeth-exposure does not only alter the perceptual properties of angry faces. In fact, angry faces with exposed teeth are usually also perceived to be more intense than angry faces with concealed teeth. For example, in one of the most frequently used databases in emotional-expression research, the KDEF database [51], angry faces with exposed teeth (32.9% of all angry faces contained in the database) obtained reliably higher intensity ratings (*M* = 5.94 on a 9-point scale) than angry faces without exposed teeth (*M* = 5.42; *t*(68) = 2.27, *p* = .026, *d*_*S*_ = 0.58). Therefore, it is hardly possible to create one set of angry faces with exposed teeth and one set of angry faces with concealed teeth that are matched in terms of intensity (particularly, if both sets contain the same identities).

This might be problematic because one clinical theory of anxiety in particular, the cognitive-motivational analysis of anxiety [28], predicts that anxiety-related differences in attentional bias towards threatening stimuli are moderated by the intensity of threat stimuli. This theory claims that anxious individuals have a hyper-sensitive valence evaluation system to the effect that even mildly negative stimuli are categorized as threatening. Thus, anxiety-related differences in attentional bias should only occur for moderately intense threat stimuli. In contrast, both anxious and non-anxious individuals should perceive highly intense negative stimuli as threatening and in turn show an attentional bias towards these stimuli. Consequently, a recent review recommends taking emotional intensity of the stimuli into account [21]. Therefore, in the present study, we also asked participants to rate the emotional intensity of the faces presented in the dot-probe task.

## Method

### Ethics statement

All participants provided informed consent prior to testing. According to the guidelines of the German Research Association (Deutsche Forschungsgemeinschaft; DFG), no ethical approval was needed for this study because it did not pose any threats or risks to the participants and participants were fully informed about the objectives of the study (http://www.dfg.de/foerderung/faq/geistes_sozialwissenschaften/index.html). The chairman of the Ethics Committee of the Faculty of Empirical Social Sciences of Saarland University confirmed that ethical approval was not needed for this study.

### Participants

Seventy-eight non-psychology university students were paid € 6 for their participation. Four participants were excluded from data analysis since their accuracy was more than 2.0 interquartile ranges below the first interquartile of the distribution. Of the remaining *N* = 74 participants, 50 were female. Their ages ranged from 19 to 35 (*M* = 24.0 years, *SD* = 3.5). All participants reported normal or corrected-to-normal vision and gave their informed consents prior to testing. Participants’ raw scores on the trait scale of the State-Trait Anxiety Inventory (STAI; [52]) ranged from 25 to 67 (*M* = 39.5, *SD* = 10.7).

### Design

We employed a 2 (*cue validity*: valid cue vs. invalid cue) × 2 (*teeth-exposure*: exposed teeth vs. concealed teeth) × STAI design with cue validity and teeth-exposure as within-subjects factors and STAI as a continuous (centered) covariate.

With regard to power considerations, we made the assumption of *r* = .30 (i.e., a “medium” effect as defined by Cohen [53]) for the STAI-cueing correlation. To detect an effect of this magnitude with a probability of 1 - β = .80 and an α-value of .05 (one-tailed), a minimum sample size of 64 participants was required; calculations were done using G.Power 3.1.3 [54].

### Materials

**Fig 1:**
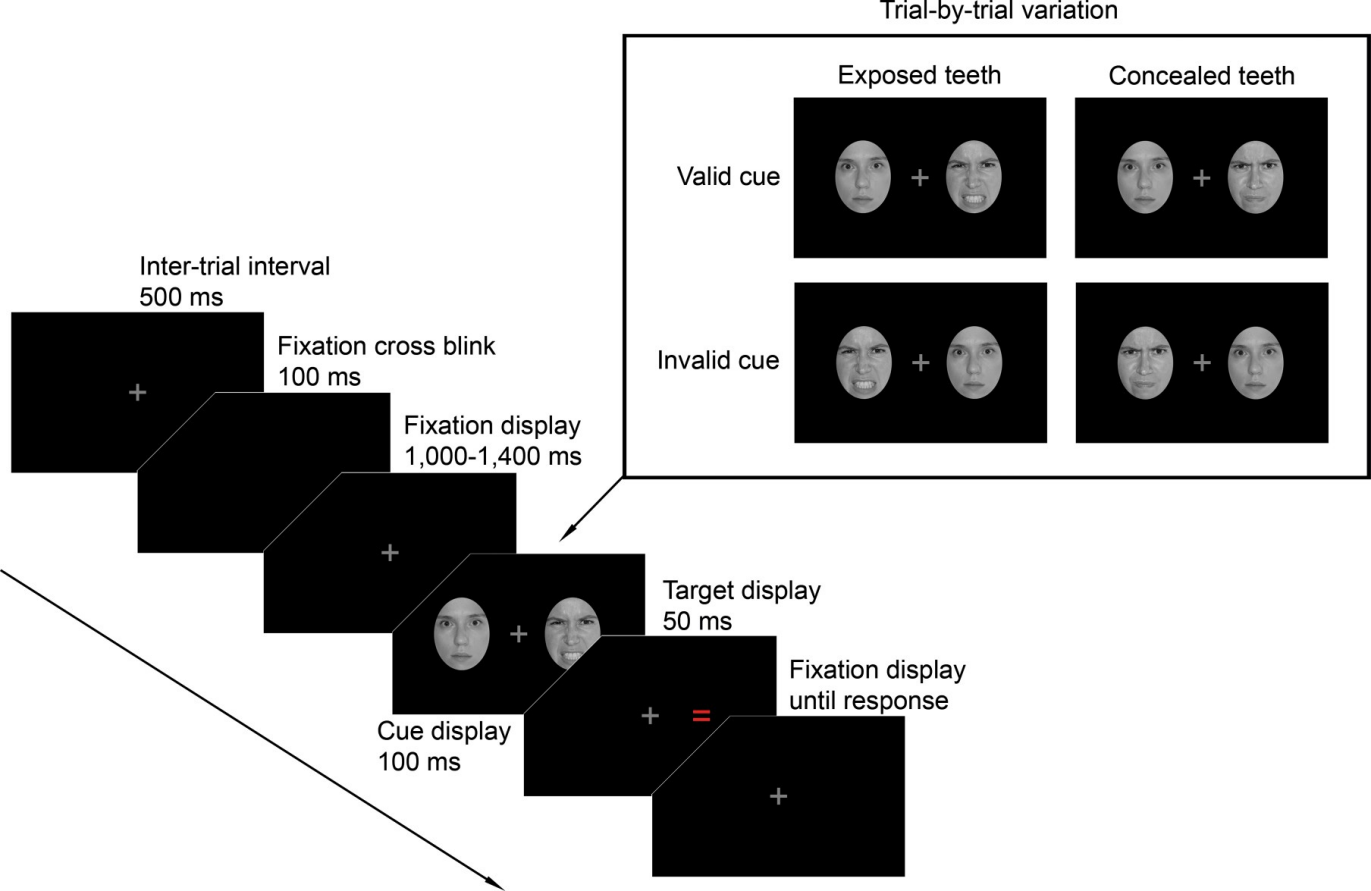
Schematic illustration of a typical trial and the design of the experiment. For the sake of visibility, proportions are not true to scale. Due to copyright issues, the photographs depicted here were not actually presented in the experiment.

Stimulus faces were taken from the NimStim database [55] since all individuals contained in this database pose each expression with both an open and a closed mouth. We selected photographs of eight female and eight male individuals displaying angry expressions with both exposed and concealed teeth. Furthermore, we selected photographs of the same 16 individuals displaying neutral expressions. All images were cropped using a standard oval shape concealing hair and external features and converted to grayscale (see Fig 1).

We chose the German version of the State-Trait Anxiety Inventory (STAI; [52]) to assess participants’ anxiety. The STAI comprises two scales (state anxiety and trait anxiety) that each contain 20 items, which are scored between 1 (low anxiety) and 4 (high anxiety). However, we decided to only administer the trait-anxiety scale of the STAI because according to a meta-analyses [25] , this is the standard in dot-probe research. Moreover, the meta-analysis shows that reliable differences between anxious and non-anxious participants regarding attentional bias to threat only occurred in those studies that relied on trait anxiety, but not in those that relied on state anxiety.

### Procedure

The study was conducted on five PCs equipped with 17" CRT monitors using a resolution of 1024 × 768 Pixels, a refresh rate of 100 Hz, and a color depth of 32 bit. The experimental routine was programmed using the Psychtoolbox-3 [56] for Matlab 2014a (Mathworks, Natick, MA).

Participants were seated in an individual testing booth approximately 65 cm from the monitor. At the beginning of the experiment, participants were presented with an instruction screen explaining the experimental procedure. Fig 1 depicts a schematic illustration of a typical trial and the design of the experiment. Throughout the study, a gray fixation cross was centrally presented on a black background to maintain participants’ focus at the central location. To indicate the beginning of a trial, the fixation cross blinked for 100 ms. The fixation cross then remained on the screen for an interval randomly chosen from the set 1,000, 1,100, 1,200, 1,300, or 1,400 ms to avoid any anticipatory effects. Two face cues were then presented laterally for 100 ms. The faces had a size of 4.5 × 6.2 cm (4.0 × 5.5°) and their center-to-center distance was 11.1 cm (9.8°). One of the face cues always wore an angry expression, the other one a neutral expression. On 50% of the trials, the angry face had exposed teeth, on the remaining trials the mouth was closed. The face cues always had the same gender, but never the same identity. Immediately after the offset of the face cues, a probe stimulus appeared at the location of one of the faces for 50 ms. Thus, the stimulus onset asynchrony, between the onset of the cues and the onset of the probe was 100 ms. We chose this SOA because we aimed to assess anxiety-related biases in shifts of covert attention. It is recommended to use short SOAs to investigate stimulus driven shifts in covert attention because they peak at 100–150 ms after stimulus onset [57]. Thus, SOAs longer than 200 ms possibly tap into shifts of overt attention [58–60]. The probe stimulus was either a “×” or a “=“ symbol and the participants’ task was to classify the stimulus as fast as possible by pressing the “t” or “v” button of a standard German QUERTZ keyboard. We chose a red-colored probe stimulus so any effects of teeth-exposure could not be attributed to the probe having a similar color to teeth (see [43]). On 50% of the trials, the probe appeared at the location of the angry face (valid cue) and on the remaining trials it appeared at the location of the neutral face (invalid cue). Each response was followed by a 500 ms inter-trial interval. If participants submitted an incorrect response or took longer than 1,500 ms, they received a 1,000 Hz warning tone lasting 500 ms via headphones. The whole procedure comprised 448 trials and lasted approximately 35 minutes. Trials were presented in a randomized order in four blocks of 112 trials, separated by self-paced breaks. At the beginning of the procedure, participants were presented with 24 training trials that were not included in data analysis.

After completing the dot-probe task, participants rated the stimuli with regard to intensity of the displayed emotional expression on a seven-point scale. At the end of the procedure, participants completed the trait scale of the German version of the STAI (see *Participants*).

## Results

Average classification accuracy was 96.2% (*SD* = 3.1). Reaction time outliers of less than 150 ms or more than 1,000 ms were excluded from data analysis (1.1% of all correct responses). After outlier removal, average individual reaction times ranged from 383 to 622 ms (*M* = 487 ms, *SD* = 49). Since the distribution of reaction times is usually skewed, we log-transformed participants’ reaction times for the statistical analyses (analyses with non-transformed reaction times essentially yielded identical results).

We calculated a 2 × 2 within-subjects ANCOVA with the factors *cue validity* (valid cue vs. invalid cue) and *teeth-exposure* (exposed teeth vs. concealed teeth), the participants’ *z*-standardized (i.e., centered) STAI scores as a covariate, and (correct) reaction times as the dependent variable. The ANCOVA revealed no significant main effects (neither of the within-subjects factors, both *F*s < 1, nor of STAI, *F* < 1), but a significant *cue validity* × STAI interaction, *F*(1, 72) = 5.08, *p* = .027, η_p_^2^ = .066, which was further moderated by a significant *cue validity* × *teeth-exposure* × STAI interaction, *F*(1, 72) = 7.63, *p* = .007, η_p_^2^ = .096 (all *F*s < 1.55 for the remaining interactions). In order to clarify the meaning of these interactions, we calculated cueing scores by subtracting participants’ reaction times to validly cued trials from reaction times to invalidly cued trials. Note that the *cue validity* × STAI interaction test reported above is equivalent to the test of the regression weight in a bivariate regression with cueing scores as dependent variable and STAI as predictor. The *cue validity* × *teeth-exposure* × STAI interaction test reported above is equivalent to the test of the regression weight of the product term in a moderated linear regression with cueing scores as dependent variable and STAI, *teeth exposure*, and their product term as predictors. Thus, the follow-up analyses to this interaction are bivariate linear regressions with cueing scores as dependent variable and STAI as predictor, separately for the *exposed-teeth* and the *concealed-teeth* condition. Since the standardized regression weight in bivariate linear regressions is equivalent to the Pearson correlation coefficient, we report the latter one for convenience.

**Fig 2:**
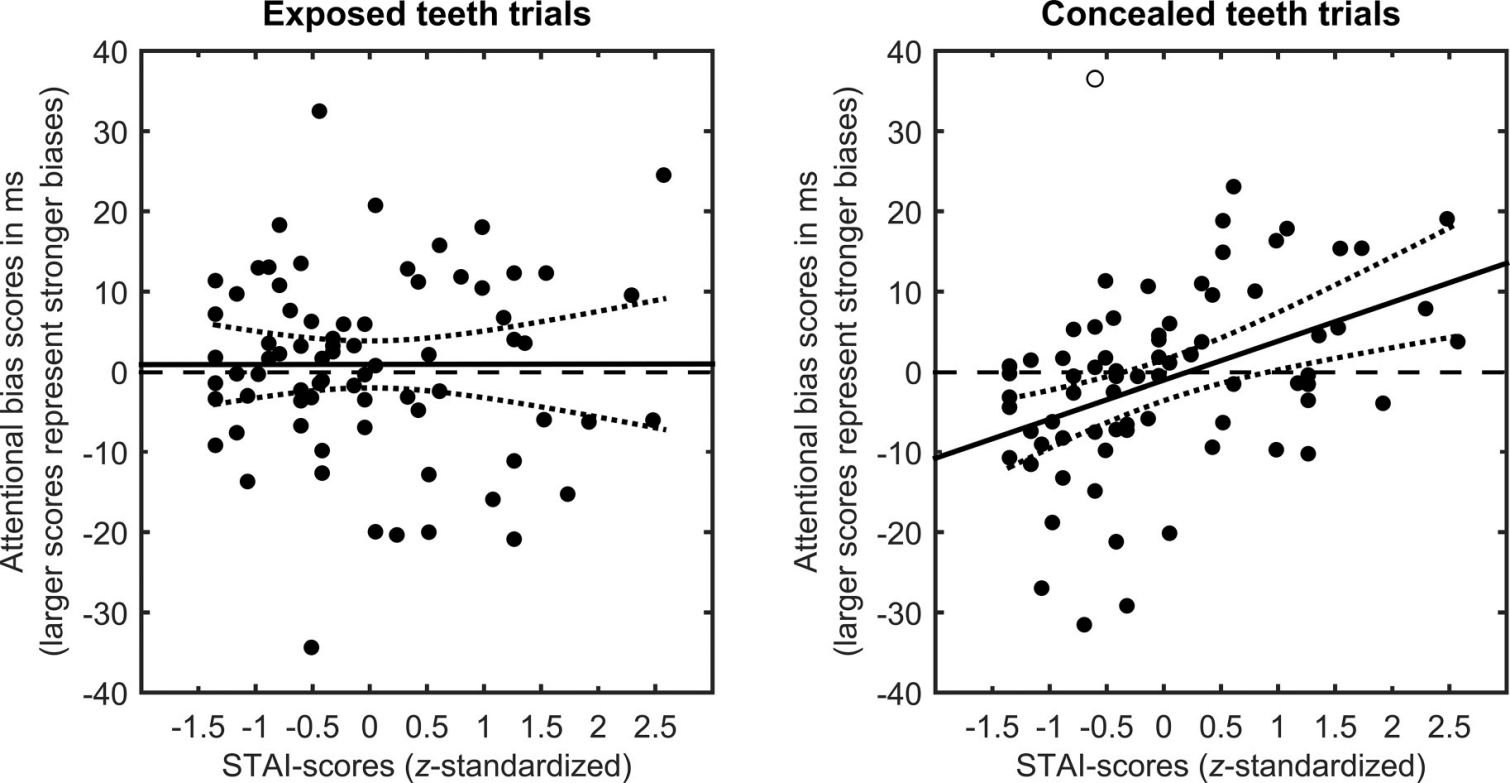
**Scatterplots illustrating the relationship between participants’ STAI-scores and their individual attentional bias scores (in ms) on exposed teeth trials (left panel) and concealed teeth trials (right panel).** The empty circle marks an excluded bivariate outlier (see text). The solid line depicts the slope of the regression, the dotted lines the 95%-confidence interval of the slope. For the sake of clarity, the attentional bias scores depicted here are based on non-transformed reaction times (RT-invalid–RT-valid).

For overall cueing scores (i.e., scores collapsed across the teeth-exposure conditions), we found a positive correlation with STAI scores, *r*(72) = .257, *p* = .027. However, as indicated by the significant interaction, there was a clear moderation by teeth exposure. For trials with exposed teeth, we found no correlation between cueing and STAI, *r*(72) = -.011, *p* = .926). For trials with concealed teeth, however, the correlation was marked, *r*(72) = .389, *p* < .001. After the exclusion of a bivariate outlier (standardized residuum *r*_s_ = 3.4), the correlation became even stronger, *r*(71) = .441, *p* < .001. Fig 2 contains scatterplots illustrating these correlations.

Unfortunately, our sample was substantially biased towards female participants. Therefore, we conducted further analyses in order to rule out the possibility that participants’ gender had a significant impact on our results. Adding participants’ gender as an additional between-subjects factor to the analyses did not yield any significant main effects or interactions involving this factor, all *F*s < 1.78, all *p*s > .186, all η_p_^2^ < .025

As expected, anger expressions with exposed teeth were rated as more intense (*M* = 6.48, *SD* = 0.31) than anger expressions without exposed teeth (*M* = 5.46, *SD* = 0.72) and this difference was significant, *t*(30) = 5.27, *p* < .001, *d*_*S*_ = 1.86. We conducted hierarchical linear modelling analyses to investigate whether the moderating effect of teeth-exposure was caused by confounded differences in intensity of the emotional expressions. We used the *lme4* and *lmerTest* packages of R 3.1.3 [61] with the significance of predictors assessed using Satterthwaite’s approximation for degrees of freedom [62]. We ran a model with cue validity (coded -1/+1), teeth exposure (coded -1/+1), *z*-standardized STAI, and all possible interaction terms as predictors and reaction times as dependent variable. We first fitted a model with random intercepts and random slopes for all predictors. In a second step, we fitted a model with random intercepts only. Because there was no significant difference in fit between models, Δχ^2^(9) < 1, the intercept-only model was accepted. Again, it yielded the significant two-way interaction of cue validity with STAI, *t* = -2.14, *p* = .032 (dfs for all reported *t*-values were > 3 × 10^4^), which was further moderated by teeth exposure, *t* = 2.20, *p* = .028. Thus, the results found by the ANCOVA (see above) could also be seen in the linear mixed model. We added to the model the mean intensity rating of the stimuli (centered), its interaction with cue validity and STAI, as well as the corresponding three-way interaction, first with random slopes, then without. Because there was no significant difference in fit between models, Δχ ^2^(9) < 1, the intercept-only model was accepted. It yielded no significant three-way interaction *cue validity* × *rating* × STAI, *t* = 0.80, *p* = .421. Thus, the emotional intensity of the stimulus faces is not the driving factor behind the *cue validity* × *teeth-exposure* × STAI interaction. We have to acknowledge, however, that due to the high correlation of teeth-exposure and emotional intensity rating, *r* = .69, the hierarchical linear modelling analyses were affected by problems of multicollinearity. Consequently, the three-way interaction *cue validity* × *teeth exposure* × STAI was reduced to a non-significant level as well, *t* = 1.03, *p* = .304, due to the redundancy of teeth exposure and intensity rating.

## Discussion

In the present dot-probe study, we replicated the well-established result that attentional bias towards angry faces is positively correlated with participants’ trait anxiety [21,25,26]. More specifically, we found that anxious participants showed a significant bias towards angry faces while non-anxious participants did not. This absence of a bias in non-anxious participants may seem surprising given that some theories of emotional attention claim that angry faces are a stimulus class of high relevance that signal imminent threat and a need for immediate action to the observer [1]. Some authors even argue that all humans should show an attentional bias towards angry faces because of evolutionary processes [3]. However, the absence of attentional bias in non-anxious participants is consistent with many previous dot-probe studies (as indicated by several meta-analyses and reviews [21,25,26]). Some recent studies investigated the question whether non-anxious participants can show an attentional bias towards threatening stimuli in the dot-probe task under specific conditions. These studies show that non-anxious participants can show an attentional bias towards threatening stimuli when current task demands require affective or social processing of the stimuli. For example, Everaert et al. [16] found a significant bias towards threatening scenes in an unselected sample when participants had to simultaneously perform a secondary task that required affective categorization, but not when the secondary task required semantic categorization. Furthermore, Wirth and Wentura [63] found an attentional bias towards angry faces in an unselected sample when target categorization required social processing of the stimuli, but not when social processing was irrelevant for target categorization.

More importantly, however, the present study showed that the correlation of trait anxiety and attentional bias towards angry faces is moderated by the exposure of teeth in the angry face cues. Specifically, we found that a significant positive correlation between participants’ trait anxiety and their individual attentional bias scores only occurred for angry faces with concealed teeth, but not for angry faces with exposed teeth. In general, this result supports our hypothesis that low-level stimulus confounds can affect the manifestation of anxiety-related biases towards task-irrelevant cue stimuli in the dot-probe task.

The main motivation of the present study were findings from another paradigm that is frequently used to measure attentional bias towards angry faces—the face-in-the-crowd paradigm. Within this paradigm, it has been shown that attentional bias towards angry faces is mainly driven by the perceptual saliency of exposed teeth (e.g., [7,33,35]). However, our results show exactly the opposite pattern. While we found an anxiety-related bias towards angry faces with concealed teeth, no such bias occurred for faces with exposed teeth.

What could be the reason that teeth-exposure causes attentional bias towards angry faces in the face-in-the-crowd paradigm but eliminates attentional bias in the dot-probe task? As already discussed in the introduction, the stimulus faces are task-relevant in the face-in-the-crowd paradigm, but not in dot-probe task. Therefore, it is possible that the general (i.e., anxiety-independent) bias towards angry faces with exposed teeth is the consequence of voluntary search strategies. It is likely that throughout the experimental procedure of a face-in-the-crowd experiment, participants learn that specific emotional expressions are characterized by salient perceptual low-level confounds. Therefore, participants might intentionally search for these salient confounds because it facilitates the task [64]. In contrast, the emotions of the face cues are task-irrelevant in the dot-probe task. Therefore, participants do not benefit from strategically attending to low-level confounds of specific emotions. Consistently, we did not find a general (i.e., anxiety-unrelated) bias towards angry faces with exposed teeth. Importantly, this result has another implication. We initially hypothesized that the mere perceptual saliency of exposed teeth (i.e., high luminance, high contrast, etc.) might be sufficient to capture participants’ spatial attention via bottom-up processes. Several studies from basic attention research have shown that highly salient stimuli (e.g., a red shape among a group of green shapes) capture attention due to their high perceptual saliency [40,41,44]. However, our results show that the saliency of exposed teeth is not sufficient to reliably capture participants’ attention.

These considerations can explain the absence of a general, anxiety-unrelated bias towards angry faces with exposed teeth. However, they cannot explain why the correlation between trait anxiety and attentional bias towards angry faces with concealed teeth did not occur for angry faces with exposed teeth. As we discussed in the introduction, one could even hypothesize that teeth-exposure increases the correlation between attentional bias and trait anxiety because anxiety is characterized by an impaired top-down control of attention [47–49]. Consequently, anxious individuals’ attention is more susceptible to bottom-up capture by salient stimuli [50]. So, what could be the reason for the absence of a correlation between trait anxiety and attentional bias towards angry faces with exposed teeth? This detail in our results is actually consistent with Mogg and Bradley’s [28] cognitive-motivational analysis of anxiety. This theory claims that trait anxiety affects the sensitivity of the valence evaluation system, to the effect that anxious individuals tag even mildly threatening stimuli with a high subjective threat value, and subsequently allocate processing resources towards it. In contrast, non-anxious individuals do not tag such stimuli with a high subjective threat value and therefore subsequently avoid the stimulus to maintain attention on current goals and to retain a positive mood state. However, with increasing threat intensity of the stimulus, non-anxious individuals should also tag the stimulus with high relevance and allocate resources to it. Thus, differences between anxious and non-anxious individuals regarding attentional bias should decrease or even vanish with increasing threat intensity of the stimulus. In an empirical test of their model, Mogg et al. [17] actually obtained comparable results to ours with mild and high threat scenes as stimuli. However, this interpretation has one shortcoming. The cognitive-motivational analysis of anxiety would predict a significant bias towards angry faces with exposed teeth in all (i.e., anxious and non-anxious) participants, which we did not find.

Thus, a different, rather simple explanation of our results seems more likely. It is possible that the perceptual properties of exposed teeth, a homogenously bright area on a darker background, are salient enough to increase the perceptual heterogeneity of the face cues. This increased heterogeneity adds noise to the data and consequently conceals the correlation between participants’ trait anxiety and attentional bias scores.

Another detail of our results warrants further discussion: A closer look at Fig 2 reveals that participants with low trait-anxiety scores actually showed a bias away from angry faces with concealed teeth. Again, this finding seems consistent with Mogg and Bradley’s [28] assumption that individuals with low trait anxiety tend to avoid mildly threatening stimuli (see above). This explanation, however, seems not very plausible in light of our short SOA of 100 ms. As pointed out by a recent review [21], the length of the SOA between cue and probe onset is an important parameter of the dot-probe task. Several researchers have advised to employ short SOAs in the dot-probe task in order to measure initial shifts in covert attention because longer SOAs might allow for multiple shifts of attention or might even tap into shifts of overt attention [58–60,65]. Since stimulus-driven shifts in covert attention peak at 100–150 ms after stimulus onset [57], it seems unlikely that within a SOA of 100 ms, participants are able to initially attend the angry face cue and subsequently avoid the angry face by shifting attention to the neutral face. An alternative explanation for the negative bias scores of non-anxious participants focuses on the meaning of neutral faces in a social context. Neutral faces are ambiguous in social meaning and ask for clarification. Thus, a bias towards neutral faces could reflect the motivation to assess the mood and intentions of the expresser. If so, the bias score might reflect the individual balance between two different tendencies, that is, to attend to clearly threat-related information versus to attend to ambiguous information. Given our results, this balance seems to be moderated by trait anxiety. Note again that dot-probe effects might reflect attentional capture or attentional dwelling (i.e., a difficulty to disengage attention from a stimulus). Thus, even if one does not accept the hypothesis of attentional capture by something ambiguous, attentional dwelling on an ambiguous stimulus is highly plausible. Of course, in case of a dwelling hypothesis, one has to assume that attention is initially directed to one of the stimuli of a dot-probe pair on a random basis.

To conclude, the present results suggest that natural low-level stimulus confounds like exposed teeth can affect attentional bias towards angry faces also in the dot-probe task where all face cues are task-irrelevant. In contrast to the face-in-the-crowd paradigm, where attentional bias towards angry faces seems to be driven by the salient properties of exposed teeth, teeth-exposure seems to interfere with the occurrence of the typically found anxiety-related attentional bias towards angry faces in the dot-probe task. Thus, future studies using emotional faces to investigate attentional bias in the dot-probe task should carefully control for natural low-level stimulus confounds like exposed teeth.
